# Roles of the Taql and Bsml vitamin D receptor gene polymorphisms in hospital mortality of burn patients

**DOI:** 10.6061/clinics/2016(08)10

**Published:** 2016-08

**Authors:** Glaucia R. Nogueira, Paula S. Azevedo, Bertha F. Polegato, Leonardo A.M. Zornoff, Sergio A.R. Paiva, Celia R. Nogueira, Natalia C. Araujo, Bruno H.M. Carmona, Sandro J. Conde, Marcos F. Minicucci

**Affiliations:** I Universidade Estadual Paulista – UNESP, Faculdade de Medicina de Botucatu, Departamento de Medicina Interna, Botucatu/SP, Brazil; II Instituto Federal São Paulo – IFSP, Campus São Roque, São Roque/SP, Brazil

**Keywords:** Vitamin D, VDR Polymorphism, Mortality, Taql, Bsml

## Abstract

**OBJECTIVE:**

The aim of this study was to evaluate the roles of the Taql and Bsml vitamin D receptor gene polymorphisms in hospital mortality of burn patients.

**METHODS:**

In total, 105 consecutive burn injury patients over 18 years in age who were admitted to the Burn Unit of Bauru State Hospital from January to December 2013 were prospectively evaluated. Upon admission, patient demographic information was recorded and a blood sample was taken for biochemical analysis to identify the presence of the Taql(rs731236) and Bsml(rs1544410) polymorphisms. All of the patients were followed over their hospital stay and mortality was recorded.

**RESULTS:**

Eighteen of the patients did not sign the informed consent form, and there were technical problems with genotype analysis for 7 of the patients. Thus, 80 patients (mean age, 42.5±16.1 years) were included in the final analysis. In total, 60% of the patients were male, and 16.3% died during the hospital stay. The genotype frequencies for the Taql polymorphism were 51.25% TT, 41.25% TC and 7.50% CC; for the Bsml polymorphism, they were 51.25% GG, 42.50% GA and 6.25% AA. In logistic regression analysis, after adjustments for age, gender and total body surface burn area, there were no associations between the Taql (OR: 1.575; CI95%: 0.148-16.745; *p=*0.706) or Bsml (OR: 1.309; CI95%: 0.128-13.430; *p=*0.821) polymorphisms and mortality for the burn patients.

**CONCLUSIONS:**

Our results suggest that the Taql and Bsml vitamin D receptor gene polymorphisms are not associated with hospital mortality of burn patients.

## INTRODUCTION

Burn injury is a type of traumatic injury that is associated with considerable morbidity and mortality 1. Burn injury is the fourth most common cause of severe injury following road accidents, falls and violence 1-2. In a systematic review, Brusselaers et al. reported that the mortality rate for burn injury ranged between 1.4 and 18%, and the major causes of death were multiple organ failure and sepsis 3. Thus, augmented systemic inflammatory responses are associated with burn patient mortality 4. To modulate the inflammatory process induced by burn injury, micronutrient supplementation, including with vitamin D, is one proposed intervention.

Vitamin D is a prohormone with a key role in balancing calcium and phosphate levels and is important for bone structure. In the last decade, the importance of vitamin D in the immune response and for inflammatory processes has been addressed. In addition, observational studies have shown an association between vitamin D deficiency and mortality in critically ill patients 5-6. Studies on vitamin D supplementation in burn patients have produced contradictory results, and whether all patients would benefit from vitamin D supplementation is unknown 7. The action of vitamin D occurs predominantly through its interaction with the vitamin D receptor (VDR). Recently, polymorphisms in the VDR gene, such as the Taql and Bsml polymorphisms, have been identified. The presence of the recessive alleles of Taql and Bsml has been associated with the prognoses of acute and chronic inflammatory diseases and could influence individual responses to vitamin D supplementation 8,9. The influence of the Taql and Bsml VDR gene polymorphisms on the mortality of burn patients has not been evaluated. Thus, the aim of this study was to evaluate the roles of the Taql and Bsml VDR gene polymorphisms in the hospital mortality of burn patients.

## MATERIALS AND METHODS

This study was approved by the Ethics Committee of Botucatu Medical School and was conducted in accordance with the Helsinki Declaration of 1975, which was revised in 1983. Written informed consent was obtained from all the patients. Burn injury patients over 18 years of age who were admitted to the Burn Unit of Bauru State Hospital from January to December 2013 were prospectively evaluated. The sample size was estimated using the following variables: 20% of the mortality risk for burn patients with a 95% confidence interval and a 10% sampling error 3. The minimum sample size required was 62 patients.

Upon admission, patient demographic information was recorded and a blood sample was obtained for biochemical analysis to identify the presence of the Taql and Bsml polymorphisms. All of the patients were followed during their hospital stay and the mortality rate was recorded.

### Laboratory data analysis

Total serum levels of sodium, potassium, albumin, creatinine and urea were measured using a dry chemistry method (Ortho-Clinical Diagnostics VITROS 950®, Johnson & Johnson). A hemogram was obtained with a Coulter STKS hematological autoanalyzer.

### Identification of VDR gene polymorphisms

DNA was isolated from frozen blood samples. Polymerase chain reaction (PCR) primers corresponding to the sequences flanking the Taql (rs731236) and Bsml (rs1544410) polymorphisms in the human vitamin D receptor gene were used. PCR was performed following a previously described method 10.

### Statistical analysis

Data are expressed as the mean±SD or the median (including the lower and upper quartiles). Statistical comparisons between groups for continuous variables were performed using Student’s t-test for parameters with a normal distribution. If the data were not normally distributed, comparisons between groups were performed using the Mann-Whitney test. Fisher’s test or the χ^2^ test was used for categorical data. Logistic regression models were adjusted for age, gender and total body surface area (TBSA) of each burn. Data analysis was performed using SigmaPlot software for Windows v12.0 (Systat Software, Inc., San Jose, CA, USA). The significance level was 5%.

## RESULTS

One-hundred and five consecutive patients were evaluated. Of these, 18 patients did not sign the informed consent form, and there were technical problems during genotype analysis for 7 patients. Thus, 80 patients (mean age, 42.5± 16.1 years) were included in the final analysis. Among these patients, 60% were male and 16.3% died during the hospital stay. The majority of the patients (47.5%) had burn injuries resulting from flame exposure. The median TBSA of the burns was 8.0% (3.0-18.8) and 47.5% of the patients were admitted to the intensive care unit.

Demographic, clinical and laboratory data according to hospital mortality are listed in Table 1. The patients who died while in the hospital were older, had higher burn TBSAs, were admitted to the intensive care unit (ICU) and had more full-thickness burns (FTBs). In addition, the patients who died had lower levels of albumin; higher white blood cell counts; and higher sodium, creatinine and urea levels. The genotype frequencies for the Taql polymorphism were 51.25% TT, 41.25% TC and 7.50% CC; for the Bsml polymorphism, they were 51.25% GG, 42.50% GA and 6.25% AA. These frequencies are consistent with those expected under the Hardy-Weinberg equilibrium. In uni- and multivariate analyses, which were adjusted for age, gender and TBSA, there were no associations between the Taql (TC+CC) (OR: 1.575; 95%CI: 0.148-16.745; *p=*0.706) and Bsml (GA+AA) (OR: 1.309; 95%CI: 0.128-13.430; *p=*0.821) polymorphisms and mortality in the burn patients (Table 2).

## DISCUSSION

The aim of this study was to evaluate the role of the Taql and Bsml VDR gene polymorphisms in the hospital mortality of burn patients. Despite the potential role of the VDR in systemic inflammation, our results suggest that these VDR gene polymorphisms are not associated with the hospital mortality of burn patients.

Brusselaers et al. 3 showed that the mortality rate of hospitalized patients with severe burn injuries in Europe ranges between 1.4 and 18%. In the present study, we calculated a mortality rate of 16.3% during hospital stay, which is in accordance with Brusselaers’ results. In addition, the three major risk factors for mortality were older age, increased TBSA and inhalation injury. The identification of risk factors for mortality is critical because it enables the identification of patients who might benefit from specific and aggressive interventions. In a recent multicenter prospective cohort study with 573 patients (347 adults), Jeschke et al. 11 showed that adults with over 40% TBSA are at high risk for morbidity and mortality. In our study, as expected, the patients who died were older, had higher burn TBSAs, more FTBs, worse kidney function and lower albumin levels. Aguayo-Becerra et al. 12 showed that burn patients with albumin levels lower than 2 g/dL have a mortality risk higher than 80%. Another variable that could influence burn patient mortality is vitamin D deficiency. However, the role of vitamin D deficiency in this scenario is not completely understood and vitamin D supplementation in burn patients has produced contradictory results.

Vitamin D deficiency is estimated to affect more than 1 billion people worldwide. The prevalence of vitamin D deficiency in burn patients is also high. Gottschlich et al. 13 showed that 45% of a study population composed of children with acute burn injuries with higher than 25% TBSAs had low values of 25-OH vitamin D. In addition, vitamin D deficiency could also occur and interfere with morbidity in chronic responses to burn injury. In post-burn scar tissue and adjacent normal skin, levels of 7-dehydrocholesterol are decreased, along with its conversion to cholecalciferol 14. In addition, burn patients are advised to avoid sun exposure to minimize scar hyperpigmentation 14. Vitamin D deficiency and upregulation of the parathyroid calcium-sensing receptor in the chronic phase of burn injury is associated with reduced bone formation and resorption, as well as osteopenia, hypocalcemia and hypoparathyroidism 15,16.

The classical actions of vitamin D include its roles in intestinal calcium absorption, bone resorption and calcium reabsorption at distal renal tubules in the presence of parathormone 17. Beyond these known effects, studies have also demonstrated associations between vitamin D deficiency and all-cause mortality 5,6. In 135 critically ill patients, Moraes et al. 6 showed that vitamin D levels lower than 12 ng/mL on ICU admission were associated with increased mortality.

Studies of vitamin D supplementation in burn patients have produced contradictory results 18-20. In adults with severe burns, Rosseau et al. 18 showed that vitamin D_3_ supplementation for 1 year during the sequelar stage of burn injury was safe and efficiently corrected hypovitaminosis D. When combined with calcium, vitamin D supplementation demonstrated positive effects on muscle health, but not on bone health. During the acute phase of burn injury, Rosseau et al. supplemented 20 burn patients and 29 healthy subjects with a single dose of 100,000 IU vitamin D_3_. The efficacy of this supplementation in raising 25(OH)D levels (and free 25(OH)D levels) was reduced or even zero in the burn patients compared to the healthy subjects. In addition, only two burn patients achieved a 30 ng/ml serum level of 25-OH vitamin D 19.

These contradictory results could be explained by previous vitamin D status, interference due to increases in serum levels of 25-OH vitamin D induced by an acute inflammatory response, and vitamin D-binding protein and VDR polymorphisms. Single nucleotide polymorphisms, such as the Taql and Bsml polymorphisms, are the most common type of genetic variation, and they could explain the differences in responses to vitamin D supplementation. However, in this study, the Taql and Bsml VDR gene polymorphisms were not associated with the hospital mortality of burn patients.

Some limitations of this study should be considered. First, we only included patients from a single medical center. Second, our sample size was relatively small. Third, other polymorphisms in the VRD gene that could influence its activity were not evaluated in this research. Additional studies on VDR polymorphisms and vitamin D supplementation should be performed in the future to better elucidate this issue.

In conclusion, our results suggest that the Taql and Bsml VDR gene polymorphisms are not associated with the hospital mortality of burn patients.

## AUTHOR CONTRIBUTIONS

Nogueira GR was responsible for study design, data collection and manuscript writing. Azevedo PS and Polegato BF were responsible for data collection. Araujo NC and Carmona BH were responsible for polymorphism determination. Paiva SA was responsible for statistical analysis. Zornoff LA, Nogueira CR, Conde SJ and Minicucci MF were responsible for study design, data interpretation and correction of the manuscript.

## Figures and Tables

**Table 1:** Demographic, clinical and laboratory data for 80 patients with burn injury according to hospital mortality.

Variables	Hospital mortality		*p*-value
	Yes (n=13)	No (n=67)	
Age (yrs.)	50.0 (37.0 – 62.5)	39.0 (29.0 – 49.0)	0.033
Male, % (n^o^)	46.2 (6)	62.7 (42)	0.421
Etiology, % (n)			
Flame	100.0 (13)	37.3 (25)	<0.001
Scald	0 (0)	40.3 (27)	
Electricity	0 (0)	17.9 (12)	
Others	0 (0)	4.5 (3)	
TBSA burn, %	38.5 (29.3-52.0)	6.5 (3.0-12.0)	< 0.001
FTB, % (n)	92.3 (12)	44.8 (30)	0.005
ICU admission, % (n)	100.0 (13)	37.3 (25)	<0.001
Hematocrit, (%)	39.4 (32.2-46.4)	38.5 (35.8-41.7)	0.461
WBC count, (x 10^3^/μL)	20680 (13510-25548)	7695 (6315-10823)	< 0.001
Creatinine, (mg/dL)	0.9 (0.8-1.6)	0.8 (0.7-0.9)	0.019
Urea, (mg/dL)	49.0 (33.5-62.0)	29.0 (21.0-36.0)	0.002
Sodium, (mmol/L)	136 (135-142)	135 (134-136)	0.010
Potassium, (mmol/L)	4.3 (3.6-4.8)	3.8 (3.4-4.3)	0.191
Albumin, (g/L)	2.6 (1.9-3.6)	3,4 (3.0-3.7)	0.012
Taql, % (n)			
TT	30.8 (4)	55.2 (37)	0.244
TC	61.5 (8)	37.3 (25)	
CC	7.7 (1)	7.5 (5)	
Bsml, % (n)			
GG	38.5 (5)	53.7 (36)	
GA	53.8 (7)	40.3 (27)	
AA	7.7 (1)	6.0 (4)	0.601

TBSA: Total body surface area (TBSA), FTB: full-thickness burn; ICU: intensive care unit; WBC: white blood cells. The data are expressed as medians (including the lower and upper quartiles) and proportions.

**Table 2:** Logistic regression models for prediction of mortality of burn patients.

Variables	Odds ratio	95%CI	*p*-value
Bsml (GA+AA)	1.858	0.551-6.270	0.318
Taql (TC+CC)	2.775	0.777-9.905	0.116
Bsml (GA+AA)*	1.309	0.128-13.430	0.821
Taql (TC+CC)*	1.575	0.148-16.745	0.706

* Adjusted by age, gender and total body surface area burn
